# Identification of the prognostic value of a 2-gene signature of the WNT gene family in UCEC using bioinformatics and real-world data

**DOI:** 10.1186/s12935-021-02215-0

**Published:** 2021-09-26

**Authors:** Yuexin Hu, Mingjun Zheng, Dandan Zhang, Rui Gou, Ouxuan Liu, Shuang Wang, Bei Lin

**Affiliations:** 1 Department of Gynecology and Obstetrics, Shengjing Hospital of China Medical University, Shenyang, China; 2 Key Laboratory of Maternal-Fetal Medicine of Liaoning Province, Benxi, China; 3 Key Laboratory of Obstetrics and Gynecology of Higher Education of Liaoning Province, Benxi, China; 4 Department of Obstetrics and Gynecology, University Hospital, LMU Munich, Marchioninistr. 15, 81377 Munich, Germany; 5 Present Address: 4th Gynecological Ward, Department of Obstetrics and Gynecology, Shengjing Hospital of China Medical University, 36 Sanhao Street, Liaoning 110004 Shenyang, People’s Republic of China

**Keywords:** UCEC, WNT2, WNT10A, Prognosis, Nomogram

## Abstract

**Background:**

The WNT gene family plays an important role in the occurrence and development of malignant tumors, but its involvement has not been systematically analyzed in uterine corpus endometrial carcinoma (UCEC). This study aimed to evaluate the prognostic value of the WNT gene family in UCEC.

**Methods:**

Pan-cancer transcriptome data of the UCSC Xena database and Genotype-Tissue Expression (GTEx) normal tissue data were downloaded to analyze the expression and prognosis of 19 WNT family genes in UCEC. A cohort from The Cancer Genome Atlas-Uterine Corpus Endometrial Carcinoma (TCGA-UCEC) was used to analyze the expression of the WNT gene family in different immune subtypes and clinical subgroups. The STRING database was used to analyze the interaction of the WNT gene family and its biological function. Univariate Cox regression analysis and Lasso cox analysis were used to identify the genes associated with significant prognosis and to construct multi signature prognosis model. An immunohistochemical assay was used to verify the predictive ability of the model. Risk score and the related clinical features were used to construct a nomogram.

**Results:**

The expression levels of WNT2, WNT3, WNT3A, WNT5A, WNT7A, and WNT10A were significantly different among different immune subtypes and correlated with TP53 mutation. According to the WNT family genes related to the prognosis of UCEC, UCEC was classified into two subtypes (C1, C2). The prognosis of subtype C1 was significantly better than that of subtype C2. A 2-gene signature (WNT2 and WNT10A) was constructed and the two significantly prognostic groups can be divided based on median Risk score. These results were verified using real-world data, and the nomogram constructed using clinical features and Risk score had good prognostic ability.

**Conclusions:**

The 2-gene signature including WNT2 and WNT10A can be used to predict the prognosis of patients with UCEC, which is important for clinical decision-making and individualized therapy for patients with UCEC.

## Background

Uterine corpus endometrial carcinoma (UCEC) is one of the most common malignancies of the female reproductive system. Epidemiological data have shown that the incidence of UCEC has increased globally in the last two decades [1], and there are expected to be 66,570 new cases and 12,940 deaths because of UCEC in the United States in 2021 [2]. Because the clinical symptoms of UCEC are predictable, most cases can be diagnosed early, and the 5-year overall survival (OS) rate is more than 90%. However, the prognosis of advanced or recurrent UCEC remains poor, with a 5-year OS rate of less than 30% for patients at International Federation of Obstetrics and Gynecology (FIGO) stage IV [3]. At present, prognostic predictors for patients with UCEC are primarily based on clinical variables such as age, FIGO stage, and pathological subtypes. Studies have shown that certain genetic or molecular changes can affect UCEC prognosis [4]. In March 2020, National Committee on Computer Network (NCCN) recommended The Cancer Genome Atlas (TCGA) molecular subtype for the first time and included it in the guidelines for diagnosis and treatment of endometrial cancer, heralding the era of genotype based precision therapy. By analyzing the genomic, transcriptome and proteomic characteristics of 373 endometrial cancer patients, TCGA divided endometrial cancer into four subtypes, namely, POLE hyper-mutation, high-mutation microsatellite instability (MSI), high-copy number type (such as p53 gene mutation) and non-specific molecular variation (NSMP). TCGA molecular subtype is important in predicting the prognosis of patients with advanced endometrial cancer and in evaluating surgical interventions to preserve reproductive function. However, TCGA molecular subtype is expensive in clinical application and high in medical costs, so we intend to explore a more convenient method to predict endometrial cancer prognosis.

The WNT gene was first cloned from mouse breast cancer induced by mouse papillomavirus, then named Int-1, and was later identified as being homologous to the wingless gene of Drosophila; therefore, these genes are collectively called WNT [5]. At present, 19 kinds of human WNT genes have been discovered, and the secretory glycoprotein encoded by WNT genes is the initiator of the WNT signaling pathway [6]. Numerous studies have demonstrated that abnormal WNT signal activation is involved in the occurrence and development of many kinds of malignant cancers such as gastric cancer [7], breast cancer [8], and colon cancer [9]. However, few studies have been conducted on WNT family genes in UCEC.

The rapid development of high throughput sequencing technology and public databases in recent years provides new ideas for data mining and a better understanding of gene function. For example, the nomogram constructed by Cheng et al. [10] can predict the OS rate of patients with UCEC using immune-related genes. The 4-gene signature constructed with autophagy-related genes based on TCGA by Zhang et al. [11] can be used to predict the prognosis of patients with UCEC. Studies using gene family genes in general to construct risk models in UCEC are relatively rare, and the value of the WNT gene family in the diagnosis and prognosis of UCEC is unclear.

In this study, the prognostic value of the WNT gene family in UCEC was comprehensively analyzed using The Cancer Genome Atlas-Uterine Corpus Endometrial Carcinoma (TCGA-UCEC) data, and the Risk score was constructed. The predictive ability of the Risk score was also validated by using data of 75 UCEC samples from Shengjing Hospital affiliated to China Medical University. The nomogram combined with clinical characteristics provides new insight into personalized prognosis prediction and clinical diagnosis of patients with UCEC.

## Materials and methods

### Data source

The pan-cancer transcriptome data of 33 cancers from the UCSC Xena database and the transcriptome data of all tissues in the Genotype-Tissue Expression (GTEx) database were downloaded. *Limma* package [12] was used to analyze the differential expression in TCGA and GTEx datasets. Univariate Cox analysis was used to identify the prognostic WNT gene in pan-cancer. The results of differential expression analysis and prognosis analysis were visualized by *pheatmap* package.

### Clinical relevance of the WNT gene family in UCEC

UCEC data were downloaded, including the clinical stage, tumor grade, pathological subtypes, TP53 mutation, age, and OS and progression-free survival (PFS) data of patients. The expression of WNT family genes in different immune subtypes and clinical subgroups was analyzed. The interaction of 19 genes was analyzed by the STRING database, the functional enrichment of genes was analyzed by Gene Ontology (GO), and the *clusterProfiler* package was used for visualization.

### Identification of UCEC molecular subtypes associated with prognosis based on WNT gene family

Nineteen WNT family genes were analyzed by univariate Cox regression analysis in UCEC, and the genes related to OS were identified. *Clusterplus* package was used for cluster analysis. The prognostic differences among different subgroups were further analyzed, and the survival curve was drawn with the *survivalROC* package. The clinical features such as clinical stage, tumor grade, tissue classification, TP53 mutation, cluster subgroup, age, and survival status were integrated, and a heatmap showing the correlation of the subtypes was drawn with the *pheatmap* package. Furthermore, principal component analysis (PCA) was carried out to compare the transcriptional spectrum of expression between different subgroups, and the ggplot2 package was used for visualization. The differentially expressed genes (DEGs) between subtypes were analyzed by the *Limma* package. The functional enrichment of gene pathways was analyzed by the gene set variation analysis (GSVA) package and visualized by the ggplot2 package. In addition, the hallmark dataset downloaded from the msigDB database was used for gene set enrichment analysis (GSEA) and further visualized by the *enrichplot* package.

### The construction of a multigene prognostic model

The TCGA-UCEC cohort was divided into a training set and a validation set in a ratio of 1:1. In the training set (training set = 272, validation set = 272), 19 genes were analyzed by univariate Cox analysis using the survival package (*p* < 0.01). Using the *glmnet* package, Lasso cox analysis was further conducted to compress the number of genes in the risk model. Lasso regression analysis [13] is a compression estimate; it helps to obtain a more refined model by constructing a penalty function, compresses some regression coefficients by forcing the sum of the absolute values of the coefficients to be less than a fixed value, and sets some regression coefficients to zero. In this study, 10 cross-validation methods were used to construct the model, and the confidence interval (CI) of each λ was analyzed.

### The construction of a nomogram using risk score and clinical features

Nomograms [14] are based on a multifactor regression analysis approach, which integrates several predictive indicators and then draws a line segment with a scale on the same plane in a certain proportion; thus, nomograms can be used to express the relationship among the variables in a prediction model. A nomogram can individually calculate the survival rate of patients with specific tumors. The hazard ratio (HR), 95% CI of the HR, and the *p* value of the risk score were analyzed by univariate and multivariate Cox regression analyses, and the nomogram was constructed with multiple predictive variables.

### Immunohistochemical analysis of WNT2 and WNT10A in patients with UCEC

We selected paraffin specimens resected from 75 patients with UCEC at Shengjing Hospital affiliated to China Medical University from 2007 to 2013. All patients were informed about the trial and signed an informed consent form, and the last follow-up date was July 20, 2020. Paraffin specimens of each UCEC tissue were fixed in 10% formalin and processed into sections at a thickness of 5 μm. Rabbit anti-human WNT2 polyclonal antibody was purchased from Solarbio (Beijing, China). Rabbit anti-human WNT10A polyclonal antibody was purchased from Proteintech (Wuhan, China). WNT2 and WNT10A expression was detected by the streptavidin-peroxidase method (SP). WNT2-positive paraffin sections of rat brain and WNT10A-positive paraffin sections of esophageal carcinoma served as positive controls. Phosphate-buffered saline (PBS) served as a negative control. The concentration of WNT2 polyclonal antibody working solution was 1:50 and that of WNT10A polyclonal antibody working solution was 1:100. The staining was carried out according to the SP kit instructions.

### Determination of the results of immunohistochemistry

The staining of brown granules in cell membrane and cytoplasm was regarded as positive. Stained cells were classified based on their color intensity using the following score system: non-stained, light yellow, brownish yellow, and dark brown, which were recorded as 0, 1, 2 and 3 points, respectively. Five fields were randomly selected from each slice under a 400-fold optical microscope to observe the score, and the average value was taken as the percentage of stained cells: < 5% is 0, 5–25% is 1, 26–50% is 2, 51–75% is 3, and > 75% is 4. The two items were multiplied to obtain the final score. The positive cell count and background evaluation were performed by two senior pathologists who were blinded to the patient data. Any objections were judged by a third pathologist. The immunohistochemical scores for WNT2 and WNT10A of each patient were substituted into the risk score formula, and the patients of the Shengjing cohort were divided into high-risk and low-risk groups according to the median risk score.

### Statistical analysis

The Chi-square test and Fisher’s exact probability test were used for the counting of data, and the Student’s *t*-test was used for comparisons between the two groups. Analysis of variance (ANOVA), Kaplan–Meier analysis, and the log-rank test were used to analyze the survival curve, and univariate and multivariate Cox regression models were used to analyze the prognostic risk factors (*p* < 0.05). All statistical analyses were performed using R software (version 3.6.1).

## Results

### Correlation of the WNT gene family and its expression with prognostic value in pan-cancer

**Fig. 1:**
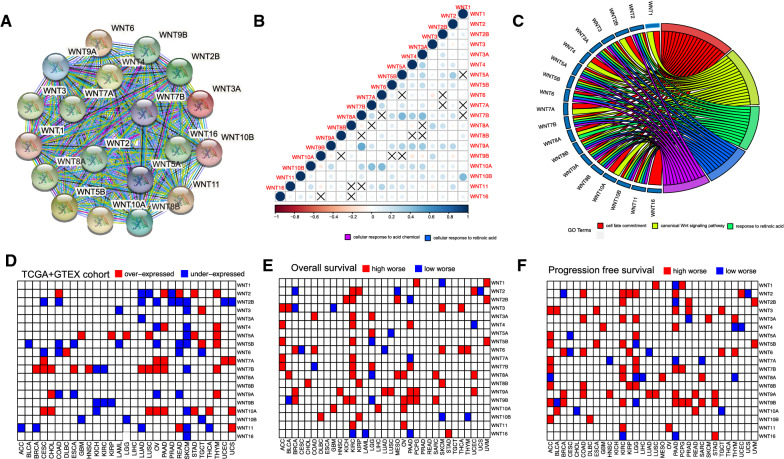
Expression and prognostic value of the WNT gene family in pan-cancer. **A** The protein-protein interaction network of the WNT gene family; **B** Correlation analysis of the WNT gene family; **C** Functional enrichment analysis of the WNT gene family; **D** WNT gene family expression in pan-cancer; **E** OS of the WNT gene family in pan-cancer; **F** PFS of the WNT gene family in pan-cancer

STRING database analysis showed the following values: number of nodes = 19, number of edges = 171, average node degree = 18, protein-protein interaction (PPI) enrichment *p* < 1.0e–16. These results indicated that there was a strong interaction among the WNT family genes (Fig. 1A). The results of correlation analysis showed that there was a significant negative correlation among most genes (Fig. 1B). Furthermore, GO functional enrichment analysis showed that most of the genes were enriched in cell fate commitment, the canonical WNT signaling pathway, and the response to the retinoic acid functional region (Fig. 1C). Differential expression analysis of the WNT gene family in pan-cancer showed that WNT2B was expressed at low levels in most tumors, WNT5A, WNT10A and WNT7B were highly expressed in most tumors, all WNT family genes were expressed at low levels in skin cutaneous melanoma (SKCM), and all WNT family genes were highly expressed in thymoma (THYM) (Fig. 1D). The relation between WNT family genes expression and OS or PFS in pan-cancer were further analyzed, and the results showed that most WNT genes were highly expressed in kidney renal clear cell carcinoma (KIRC) and adrenocortical carcinoma (ACC), suggesting poor prognosis. WNT9A, WNT8B, and WNT3A were highly expressed in most tumors with poor prognosis (Fig. 1E, F).

### Correlation among the WNT gene family, immune subtype, and TP53 mutation

**Fig. 2:**
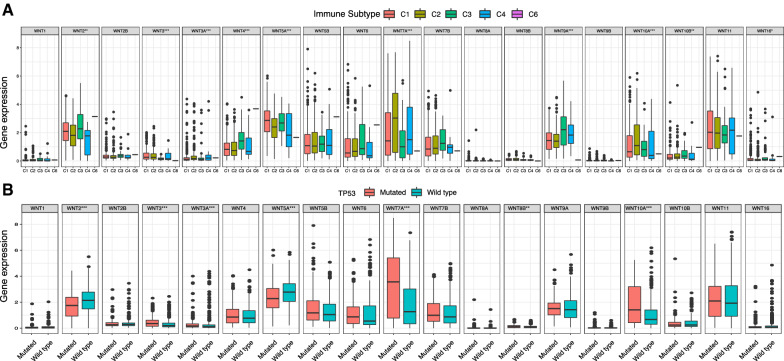
**A** Correlation between the WNT gene family and immune subtypes; **B** Correlation between the WNT gene family and TP53 mutations

In previous studies, immune-genomic analysis was performed on more than 10,000 tumor samples from 33 cancer types from TCGA. In cross-tumor studies, six immune subtypes including C1 (wound healing), C2 (INF-r-dominant), C3 (inflammation), C4 (lymphocyte depletion), C5 (immunologically silent), and C6 (TGF-β-dominant) were identified [15]. In view of the immunological silence of the C5 subtype, we analyzed the other five immune subtypes. The results showed that the expression of WNT2, WNT3, WNT3A, WNT4, WNT5A, WNT7A, WNT9A, WNT10A, WNT10B, and WNT16 were significantly different in different subtypes (Fig. 2A). The TP53 tumor suppressor gene is the most commonly altered gene in human tumors. Studies have shown that p53 mutations are widespread in UCEC, especially in type II UCEC, and TP53 expression increases gradually with disease progression [16–18]. It has been suggested that TP53 mutation may be an independent prognostic factor for endometrial cancer. Therefore, the correlation between the WNT gene family and TP53 mutation was analyzed. The results showed that the WNT2, WNT3, WNT3A, WNT5A, WNT7A, WNT8B, and WNT10A genes were significantly associated with TP53 mutation (Fig. 2B). These findings suggest that TP53 mutation may be involved in malignant tumor progression via activation of the WNT/β-catenin signaling pathway.

### Relation between the WNT gene family and clinicopathological parameters of UCEC

**Fig. 3:**
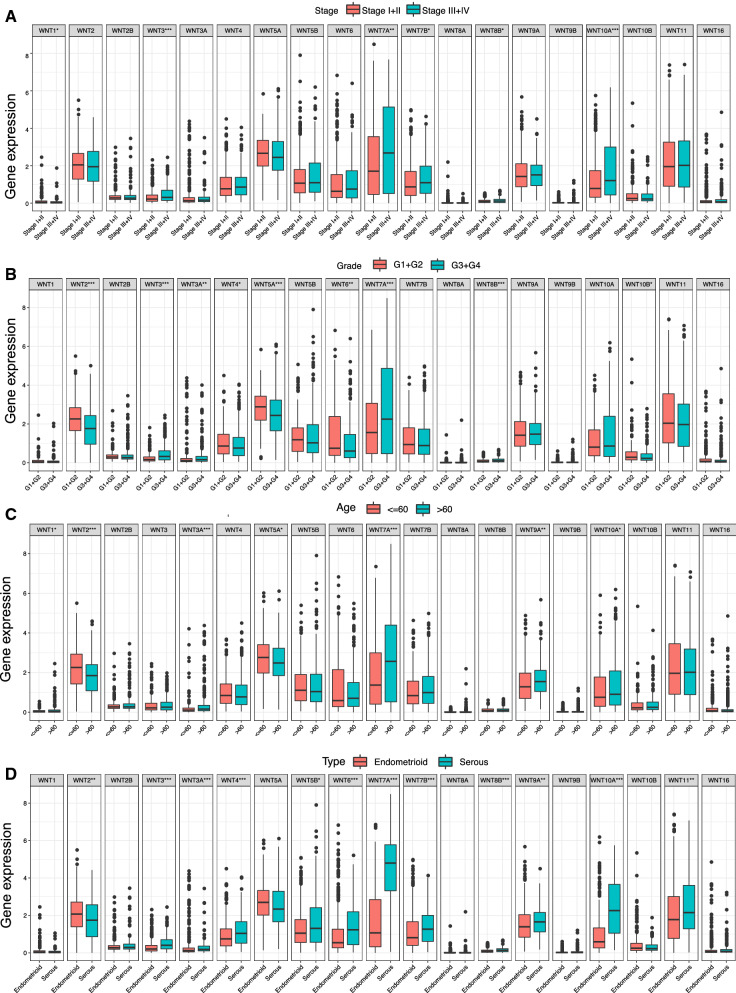
Relationship between the WNT gene family and clinicopathological parameters of UCEC.**A** Correlation between expression of WNT family genes and FIGO stage; **B** Correlation between expression of WNT family genes and grade; **C** Correlation between expression of WNT family genes and age; **D** Correlation between expression of WNT family genes and tissue subtypes

Further analysis of the correlation between the WNT gene family and clinicopathological parameters of UCEC showed that the WNT1, WNT3, WNT7A, WNT7B, WNT8B, and WNT10A genes were expressed at significantly different levels at different tumor stages (Fig. 3A). There were significant differences in the expression levels of WNT2, WNT3, WNT3A, WNT4, WNT5A, WNT6, WNT7A, WNT8B, and WNT10B among different tumor grades (Fig. 3B). The expression of the WNT1, WNT2, WNT3A, WNT5A, WNT7A, WNT9A, and WNT10A genes was significantly different in different age groups (Fig. 3C). There were significant differences in WNT2, WNT3, WNT3A, WNT4, WNT5B, WNT6, WNT7A, WNT7B, WNT8B, WNT9A, WNT10A, and WNT11 expression among different histological types (Fig. 3D).

### Molecular subtype and functional enrichment analysis based on WNT family genes

**Fig. 4:**
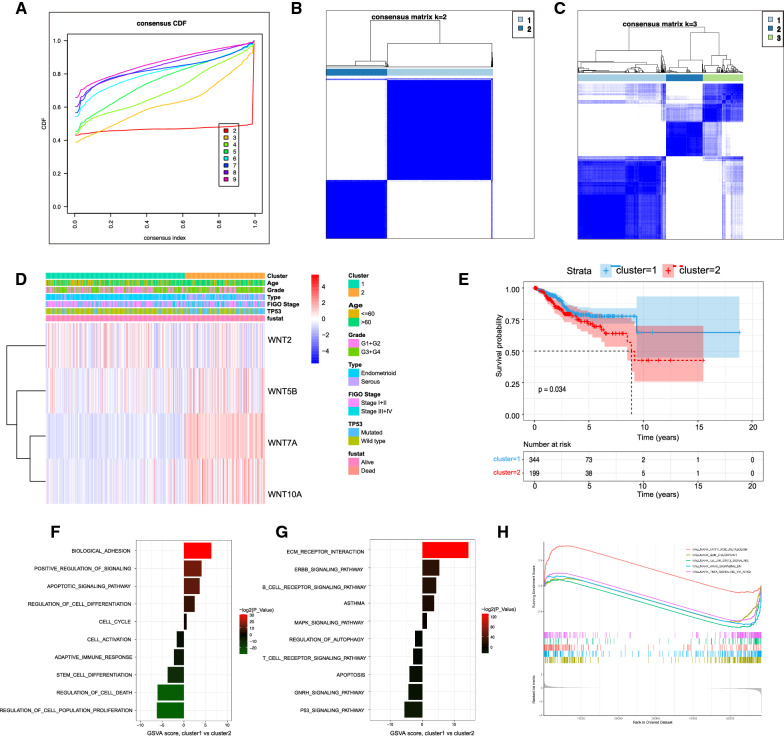
Molecular typing of UCEC based on the WNT gene family.**A** Cumulative distribution function (CDF) curve; different colors represent different cluster numbers. The horizontal axis represents the consensus index, the vertical axis represents the CDF, and a bigger AUC indicates better clustering; **B** Heatmap of sample clustering at consensus k = 2; **C** Heatmap of sample clustering at consensus k = 3; **D** Gene expression heatmap of significantly prognostic genes in two subtypes. Red represents high expression and blue represents low expression; **E** Survival curve between different cluster groups; **F** Enrichment histogram of DEGs between cluster1 and cluster2 in biological process enrichment; **G** Enrichment histogram of DEGs between cluster1 and cluster2 in KEGG; **H** Enrichment analysis of DEGs in a hallmark gene set

Four genes including WNT2, WNT5B, WNT7A, and WNT10A were identified by univariate Cox regression analysis in all TCGA datasets, and cluster analysis was performed using the *Clusterplus* package. The results showed that WNT2, WNT5B, WNT7A, and WNT10A were related to the prognosis of UCEC. In the TCGA dataset, the cumulative distribution function (CDF) curve of the WNT family genes showed that k = 3 seems to be an appropriate choice for cluster, but the clustering effect was more stable when k = 2 (Fig. 4A–C). Patients with UCEC were therefore divided into C1 and C2 subtypes. The DEGs between the C1 and C2 subtypes were analyzed using the *Limma* package [12], and a heatmap was drawn (Fig. 4D). Survival analysis showed that there was a significant difference between the C1 and C2 subtypes. The prognosis of the C1 subtype was significantly better than that of the C2 subtype (Fig. 4E). The functional enrichment of DEGs between the two subtypes was analyzed. The results of biological process enrichment are shown in Fig. 4F, which were mainly enriched in BIOLOGICAL_ADHESION, POSITIVE_REGULATION_OF_SIGNALING, APOPTOTIC_SIGNALING_PATHWAY, and REGULATION_OF_CELL_POPULATION_PROFLIFERATION. The results of Kyoto Encyclopedia of Genes and Genomes (KEGG) pathway enrichment analysis are shown in Fig. 4G; these were mainly enriched in ECM_RECEPTOR_INTERACTION, ERBB_ SIGNALING_ PATHWAY, P53_SIGNALING_PATHWAY, and GNRH_ SIGNALING_PATHWAY. In addition, the results of GSEA analysis using the hallmarker gene set showed that the DEGs were enriched in G2M checkpoints, IL6_JAK_STAT3 pathway, and KRAS pathway (Fig. 4H).

### Construction and validation of a 2-gene signature

**Fig. 5:**
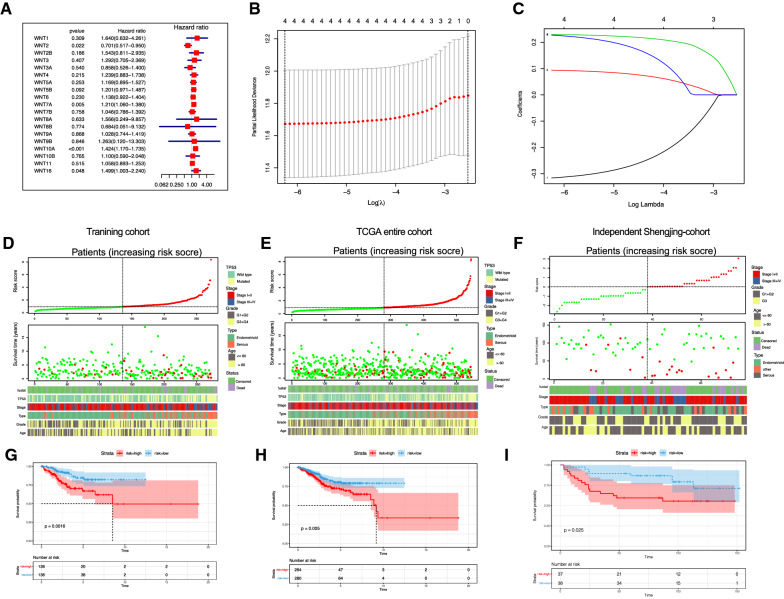
Construction and validation of the 2-gene signature based on the WNT gene family.**A** Univariate cox analysis of WNT family genes in the TCGA-UCEC cohort training set; **B** The confidence interval under each lambda; **C** The changing trajectory of each independent variable. The horizontal axis represents the log value of the independent variable lambda, and the vertical axis represents the coefficient of the independent variable; **D** Risk score, survival time, survival status, and 2-gene signature expression in the TCGA training set; **E** Risk score, survival time, survival status, and 2-gene signature expression in all TCGA datasets; **F** Risk score, survival time, survival status, and 2-gene signature expression in the Shengjing cohort; **G** The KM survival curve distribution of the 2-gene signature in the TCGA training set; **H**. he KM survival curve distribution of the 2-gene signature in all TCGA datasets; **I** The KM survival curve distribution of the 2-gene signature in the Shengjing cohort

We sought to further explore the prognostic role of WNT family genes in UCEC. Univariate Cox analysis of 19 genes using the survival package showed that WNT2, WNT7A, WNT10A, and WNT16 were significantly correlated with OS (*p* < 0.05) (Fig. 5A). The change trajectory of independent variables showed that the number of independent variable coefficients tending towards zero gradually increased with the gradual increase of lambda (Fig. 5C). The 10-fold cross-validation method was used to build the model, and the CI under each lambda was analyzed. The results showed that when log (lambda) = – 6.3, the model is optimal; we therefore chose the four genes as target genes when log (lambda) = – 6.3 (Fig. 5B). Multivariate Cox regression analysis showed that the WNT2 and WNT10A genes were still significantly associated with prognosis. The risk model of the two genes is as follows: risk score = − 0.333*WNT2 + 0.337*WNT10A.

The risk score of each patient was calculated according to this formula. The results showed that with an increasing risk score, the age of the patients was older, the tumor stage was later, the tissue grade was higher, there were more TP53 mutations, and the survival status was worse (Fig. 5D–E). Patients were divided into high-risk and low-risk groups according to the median risk score value. Survival analysis showed that there were significant differences between the high-risk and low-risk groups, both in the training set and in all datasets (Fig. 5G, H).

**Fig. 6:**
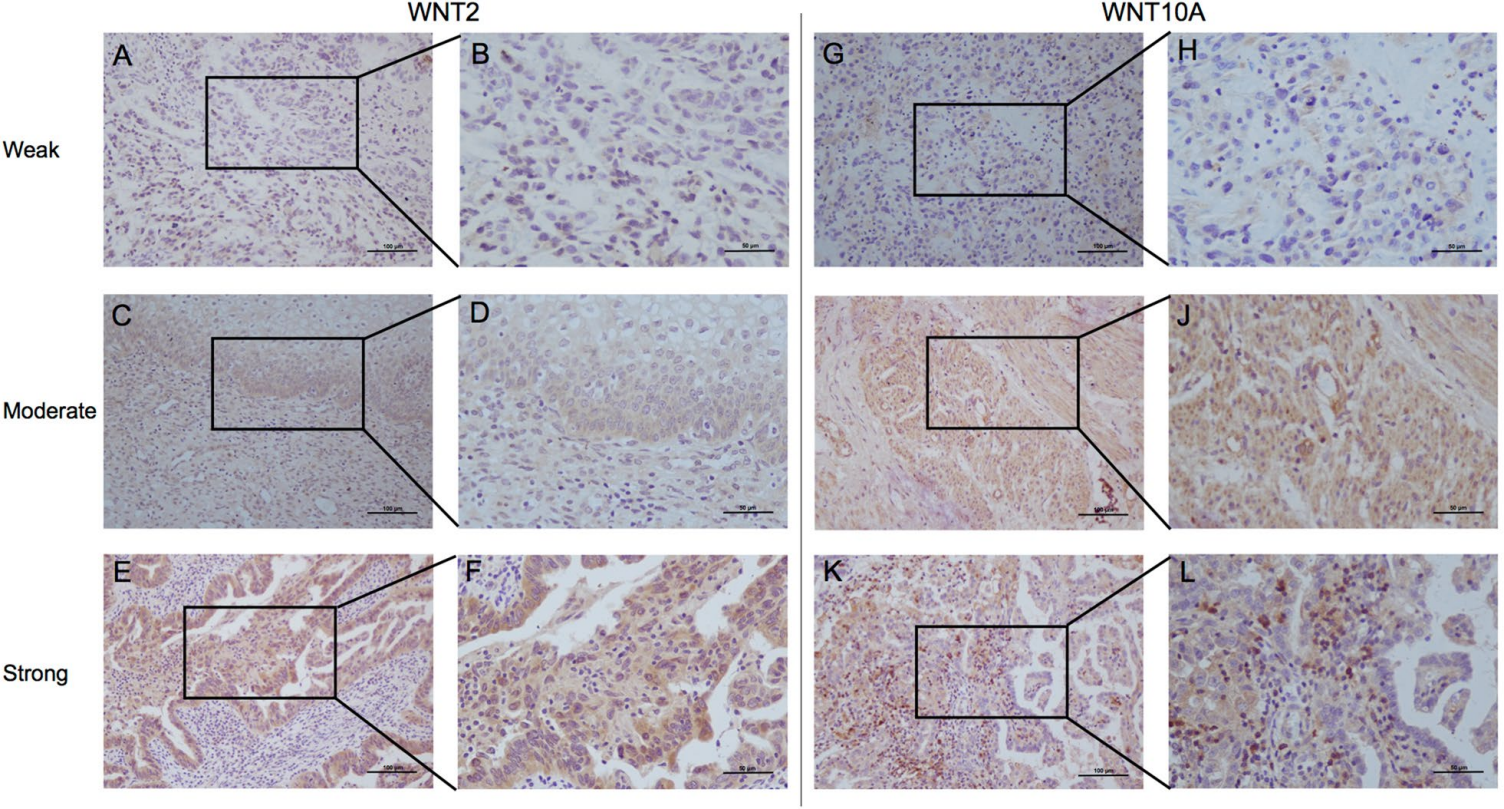
Expression of WNT2 and WNT10A in UCEC.** A**–**B** Representativeness of weak WNT2 staining in UCEC (200× and 400×); **C**–**D** Representativeness of moderate WNT2 staining in UCEC (200× and 400×); **E**–**F** Representativeness of strong WNT2 staining in UCEC (200× and 400×); **G**–**H** Representativeness of weak WNT10A staining in UCEC (200× and 400×); **I**–**J** Representativeness of moderate WNT10A staining in UCEC (200× and 400×); **K**–**L** Representativeness of strong WNT10A staining in UCEC (200× and 400×)

The Shengjing UCEC cohort was used to further verify the prognostic ability of the risk score in the real world. Firstly, immunohistochemistry was used to evaluate WNT2 and WNT10A expression in patients with UCEC, and the representative weakly and strongly stained images were selected, as shown in Fig. 6. The risk score of each patient was calculated according to the formula and the risk score coefficient in the training set. The patients were divided into high-risk and low-risk groups according to the median risk score value. The results of survival analysis showed that there were still differences in survival outcomes between the high-risk and low risk group in an external independent cohort (Fig. 5F).

### Correlation between risk score and clinical subgroups

**Fig. 7:**
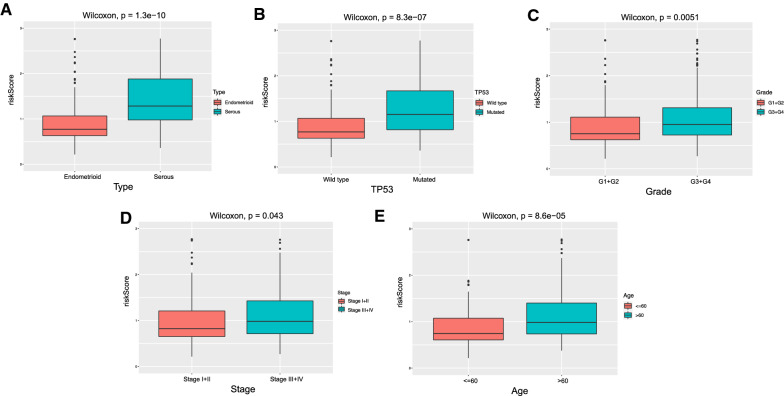
Correlation analysis of risk score among different clinical subgroups.**A** Risk score correlation among histological types; **B** Risk score correlation between TP53 mutation and wild type; **C** Risk score correlation between different grades; **D** Risk score correlation between different stages; **E** Risk score correlation between different age groups

The correlation between clinical subgroups and risk score was further analyzed. The results showed that the risk score of patients with age > 60, stage III + IV cancer, serous type, mutated type was significantly higher than that of other patient groups, indicating that our signature can facilitate subgroup diagnosis according to different clinical features (Fig. 7).

### Nomogram and its clinical diagnostic ability

**Fig. 8:**
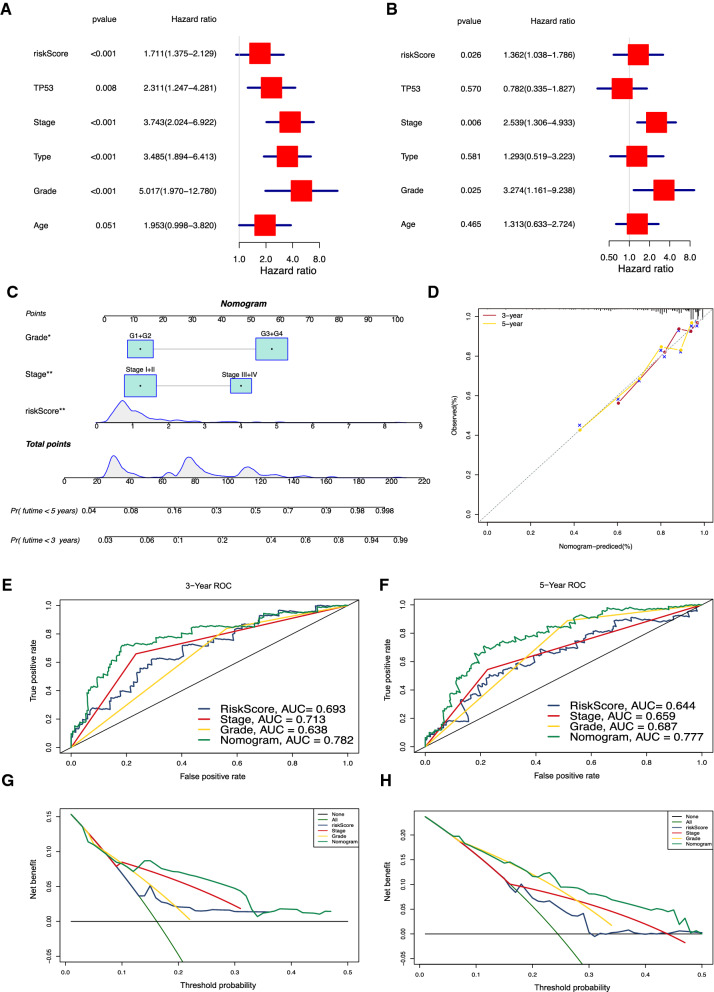
Clinical diagnostic value of the 2-gene signature. **A** Forest map of univariate Cox analysis; **B** Forest map of multivariate Cox analysis; ** C** Nomogram for predicting the 3- and 5-year OS of patients; **D** The 3- and 5-year calibration curves of the nomogram; **E** The 3-year ROC of the nomogram; **F** The 5-year ROC of the nomogram; **G** The 3- year DCA curves of the nomogram; **H** The 5- year DCA curves of the nomogram

We further analyzed the relationship between risk score and other variables and the prognosis of patients with UCEC. Forest maps can simply and intuitively show the statistical results of different factors, as shown in(Fig. 8A, B. The risk score (HR = 1.362, *p* = 0.026), FIGO stage (HR = 2.539, *p* = 0.006), and grade (HR = 3.274, *p* = 0.035) were significantly correlated with survival and were independent risk factors for the prognosis of patients with UCEC. A nomogram was constructed with the stage, grade, and risk score. As observed from the results of the model, the risk score has the greatest influence on predicting the survival outcome, indicating that the risk model based on the WNT2 and WNT10A genes can better predict the prognosis of endometrial cancer (Fig. 8C). The performance of the 3- and 5-year nomograms can be displayed using a calibration plot. It showed that the nomogram performed well in predicting the prognosis of UCEC (Fig. 8D). The 3–5-year area under the curve (AUC) of the nomogram was also the largest when compared with the other clinical variables (Fig. 8E, F). Decision curve analysis (DCA) was used to evaluate the clinical effectiveness of the model, and the results showed that the nomogram had the best net benefit for predicting patient survival (Fig. 8G, H).

These results showed that compared with the nomogram constructed using a single clinical factor, the nomogram comprising risk score and significant variables constructed using a combined model is the best for predicting UCEC patients’ survival. This model may therefore be helpful for clinical decision-making and personalized treatment.

## Discussion

According to the traditional classification, UCEC can be divided into type I and type II, based on different origins, pathogenesis, and genetic characteristics [19]. Type I UCEC is estrogen-dependent and usually has a good prognosis. At the molecular level, type I endometrial cancer is associated with mutations in genes such as PTEN, KRAS, ARID1A, PIK3CA, and CTNNB1 and microsatellite instability (MSI). Abnormal changes in the genes may result in abnormal PTEN-PIK3/AKT/mTOR signaling pathways, which can lead to tumor development [20]. Type II UCEC is non-estrogenic and has a poor prognosis. At the molecular level, type II endometrial cancer is characterized by p53 mutations and HER2 overexpression [21]. At present, clinicopathological features of UCEC such as pathological type and grade, FIGO stage, myometrial invasion, and lymphatic vascular invasion (LVSI) are used for risk stratification and diagnostic and therapeutic decision-making [22]. However, due to the heterogeneity of UCEC, several studies have focused on molecular changes that occur at the genetic level in UCEC [23, 24]. Changes in the WNT/β-catenin pathway are found in about 65% of the patients with UCEC [25]. The WNT/β-catenin pathway is involved not only in the regulation of normal endometrium but also in endometrial hyperplasia and carcinogenesis [26]. As the promoter of the WNT/β-catenin signaling pathway, the WNT gene family has attracted much attention. By analyzing the expression profiles and follow-up data of 19 molecules encoded by the WNT gene family in different types of tumors, the results showed that some WNT gene family genes can be used as prognostic indicators for patients. For example, WNT2 overexpression in colorectal cancer and hepatocellular carcinoma indicates poor prognosis [27, 28]. Additionally, WNT3A overexpression in esophageal squamous cell carcinoma and hepatocellular carcinoma indicates poor prognosis [29, 30].

Despite these findings, few studies have been conducted on UCEC thus far. Based on existing evidence, we speculated that the WNT gene family also plays an important role in predicting prognosis and risk stratification of UCEC. Since multiple factors may influence the expression of a single gene, it is not sufficient to independently predict prognosis in patients with UCEC. Compared with a single biomarker, the combined model constructed with multiple related genes is more accurate in predicting prognosis and is of great significance for individual diagnosis and treatment and for predicting UCEC patient prognosis [11, 31, 32]. Therefore, this study comprehensively evaluated the prognostic value of the WNT gene family in UCEC for the first time.

By analyzing the expression and prognosis of WNT family genes in pan-cancer, we found that WNT9A was overexpressed in most tumors and indicated poor prognosis. This is consistent with previous research results [33]. In UCEC, both PFS and OS were shortened in patients with high WNT2 expression, indicating poor prognosis. Studies have shown that the WNT/β-catenin signaling pathway is involved in tumor cell immune escape. In a teratoma model, increased WNT expression was related to impaired immune cell recruitment and decreased T cell and B cell infiltration, suggesting that the immune surveillance function was impaired [34]. Further analysis of the correlation between WNT family genes and immune subtypes showed that there were significant differences in the expression levels of WNT2, WNT3, WNT3A, WNT4, WNT5A, WNT7A, WNT9A, WNT10A, WNT10B, and WNT16 among different immune subtypes. Studies have shown that when WNT2 is knocked down, IL-8 expression is increased in epithelial cells [35]. WNT5a has dual effects on the tumor microenvironment; it can activate the autocrine ROR1/Akt/P65 pathway and promote immune cell inflammation and chemotaxis. WNT5a can also specifically activate the TLR/MyD88/p50 pathway in bone marrow monocytes and promote the synthesis of anti-inflammatory cytokines such as IL-10 and the tolerance phenotype, thus forming an immunosuppressive tumor microenvironment [36]. Although the mechanism of the WNT family genes involved in immune regulation is not clear, our results suggest that WNT family genes may be used as important markers to distinguish immune subtypes.

Further analysis of the correlation between the WNT gene family and the clinicopathological parameters of UCEC showed that WNT7A was significantly different in different clinical stages, tumor grades, age groups, and histological types. Some studies have shown that WNT7A is overexpressed in UCEC and indicates a poor prognosis [37]. However, its mechanism in the occurrence and development of UCEC still needs to be explored further.

We used the TCGA-UCEC cohort to analyze 19 WNT genes by univariate and multivariate Cox regression analyses and Lasso regression analysis. The results showed that WNT2 and WNT10A were significantly correlated with prognosis. The risk score of the 2-gene signature was constructed, and the patients were divided into high-risk and low-risk groups according to the median risk score. Although previous studies have evaluated the prognostic value of the WNT gene family in prostate cancer [38] and hepatocellular carcinoma [28], these studies are only included in the TCGA cohort. The main ethnic groups in the TCGA cohort are black and white, and there is a lack of data regarding the Asians. This study is the first time that a real-world cohort has been applied for studying UCEC, and thus its prognosis prediction is more reliable. Furthermore, the meaningful clinical features of multivariate Cox analysis were combined with the risk score to construct a nomogram. The results of the calibration map and DCA curve show that our model has a good ability to predict UCEC patient prognosis. Compared with the other prognostic models of UCEC [39, 40], our model contains fewer genes and is more convenient to use in clinical practice.

Despite these benefits, our research still has some limitations. Our study is a retrospective study that still needs to be verified by a prospective study in the future. Secondly, although we have included real-world data for verification, the sample size is small, and further research with a larger sample size is needed in the future.

## Conclusions

Two genes (WNT2 and WNT10A) significantly related to prognosis of UCEC were identified by comprehensively analyzing the prognostic value of the WNT family and risk score was constructed. Patients with UCEC can be divided into high-risk and low- risk groups according to the risk score, and the high-risk group has a poor prognosis. The 2-gene signature provides new avenues for prognosis prediction and clinical decision-making in UCEC.

## Data Availability

The datasets used and analysed during the current study are available from the corresponding author on reasonable request.

## Abbreviations

UCEC: Uterine corpus endometrial carcinoma
OS: Overall survival
FIGO: International Federation of Obstetrics and Gynecology
NCCN: National Committee on Computer Network
TCGA: The Cancer Genome Atlas
MSI: Mutation microsatellite instability
NSMP: Non-specific molecular variation
GTEx: Genotype-Tissue Expression
PFS: Progression-free survival
GO: Gene Ontology
PCA: Principal component analysis
DEGs: Differentially expressed genes
GSEA: Gene set enrichment analysis
GSVA: Gene set variation analysis
CI: Confidence interval
HR: Hazard ratio
SP: Streptavidin-peroxidase
PBS: Phosphate-buffered saline
ANOVA: Analysis of variance
PPI: Protein–protein interaction
THYM: Thymoma
SKCM: Skin Cutaneous Melanoma
KIRC: Kidney renal clear cell carcinoma
ACC: Adrenocortical carcinoma
CDF: cumulative distribution function
KEGG: Kyoto Encyclopedia of Genes and Genomes
AUC: Area under the curve
